# Supplementary figures and images for: Germline loss in C. elegans enhances longevity by disrupting adhesion between niche and stem cells
Source: EMBO J. 2024 Jul 25;43(18):9. doi: 10.1038/s44318-024-00185-3 (PMC11405865; doi:10.1038/s44318-024-00185-3)

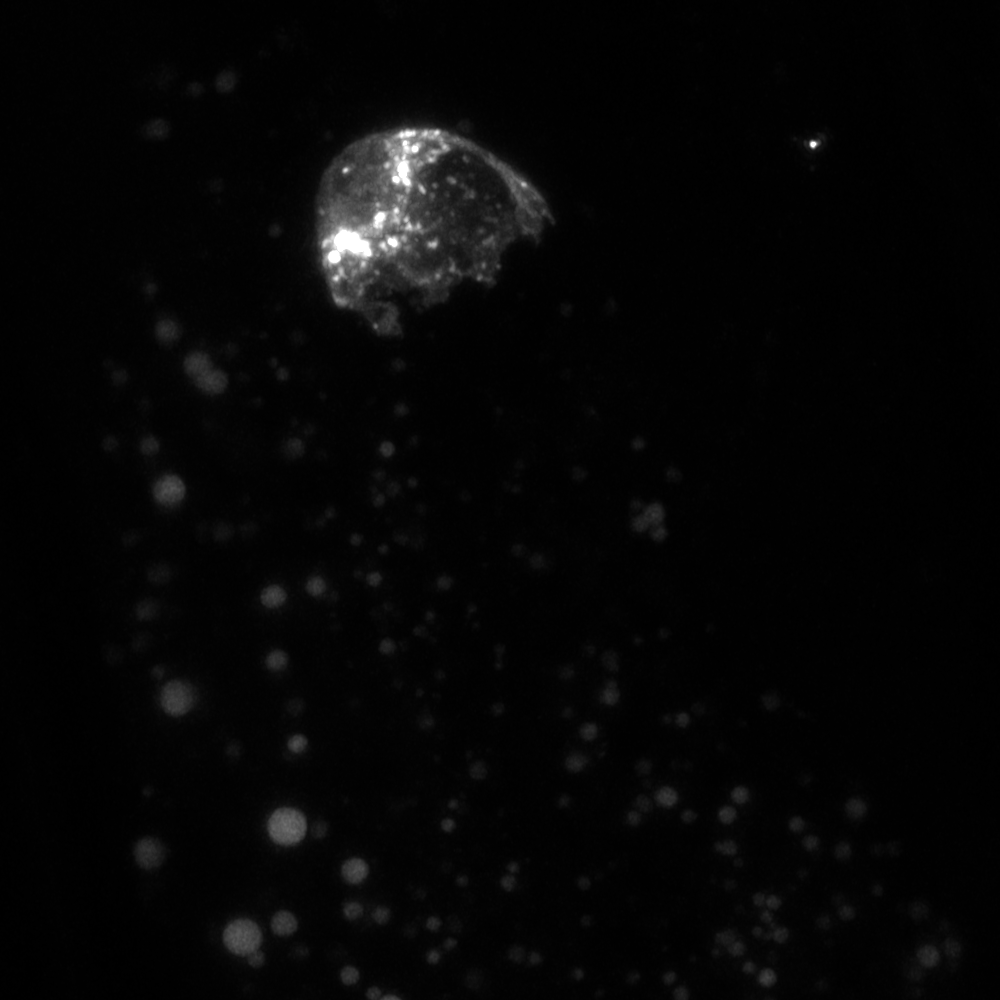

Supplement: Supplementary file 8 — Source data Fig. 3 [file 44318_2024_185_MOESM8_ESM.zip › 3B/Fig 3B glp-1 shown slice.tif]

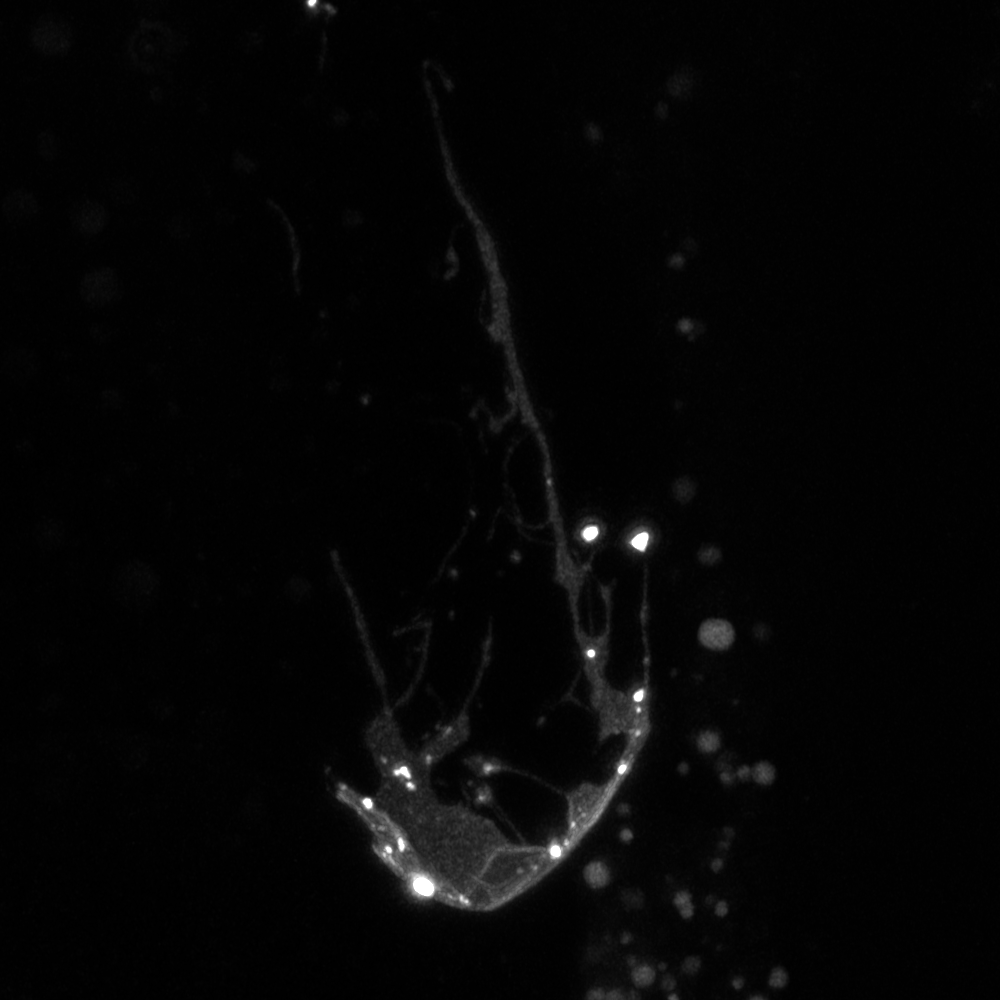

Supplement: Supplementary file 8 — Source data Fig. 3 [file 44318_2024_185_MOESM8_ESM.zip › 3B/Fig 3B WT shown slice.tif]

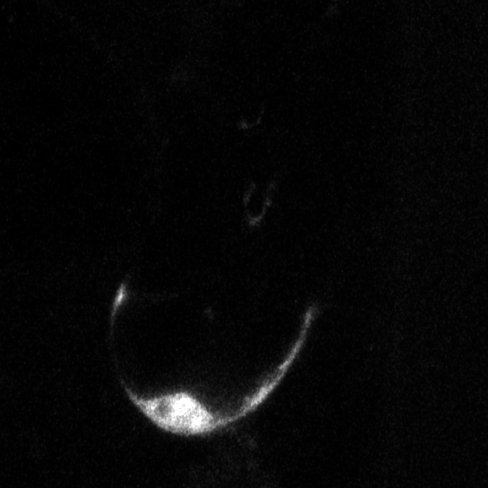

Supplement: Supplementary file 9 — Source data Fig. 4 [file 44318_2024_185_MOESM9_ESM.zip › 4B/Fig 4B hmr-1 RNAi shown slice.tif]

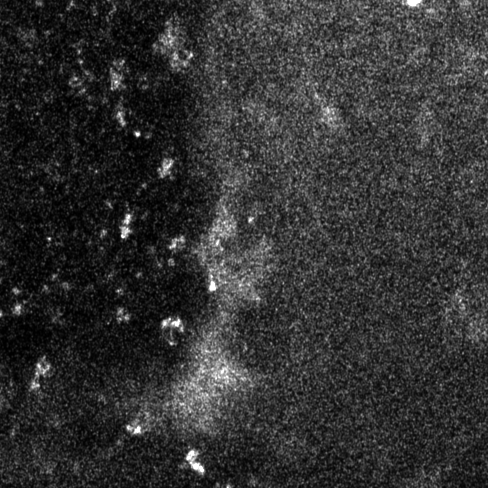

Supplement: Supplementary file 9 — Source data Fig. 4 [file 44318_2024_185_MOESM9_ESM.zip › 4B/Fig 4B hmr-1 RNAi z-stack.tif]

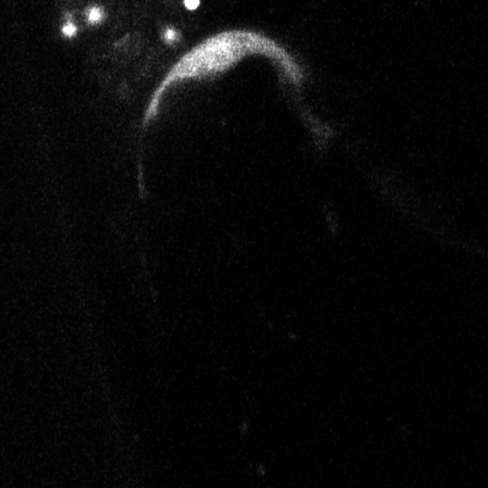

Supplement: Supplementary file 9 — Source data Fig. 4 [file 44318_2024_185_MOESM9_ESM.zip › 4B/Fig 4B luc2 RNAi shown slice.tif]

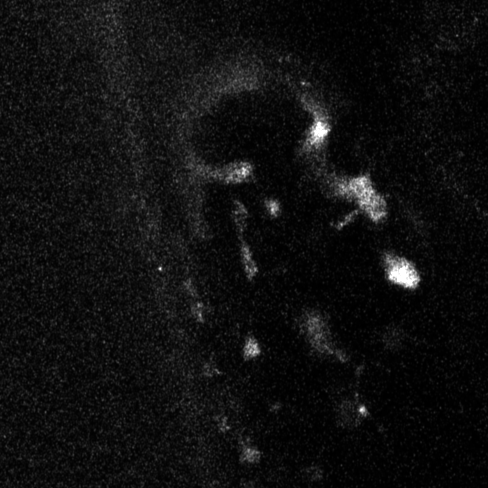

Supplement: Supplementary file 9 — Source data Fig. 4 [file 44318_2024_185_MOESM9_ESM.zip › 4B/Fig 4B luc2 RNAi z-stack.tif]

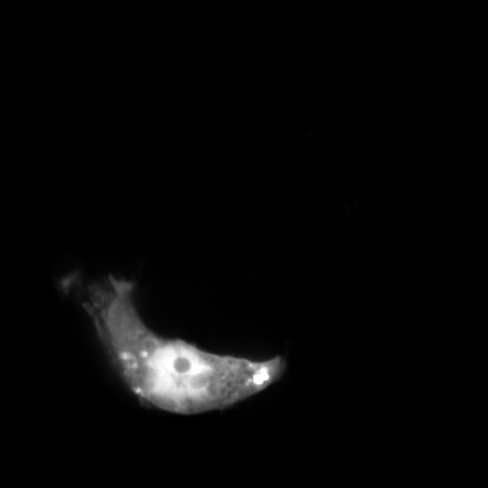

Supplement: Supplementary file 9 — Source data Fig. 4 [file 44318_2024_185_MOESM9_ESM.zip › 4A/Fig 4A glp-1 shown slice.tif]

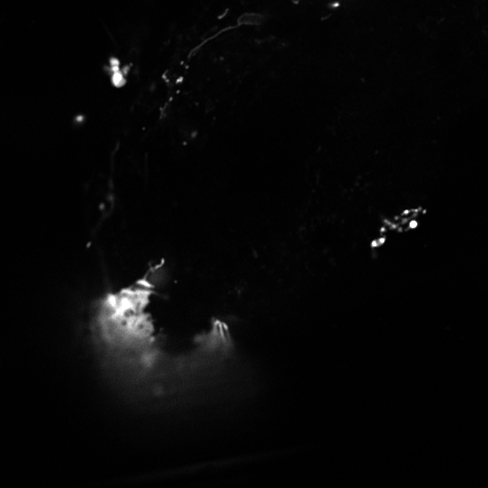

Supplement: Supplementary file 9 — Source data Fig. 4 [file 44318_2024_185_MOESM9_ESM.zip › 4A/Fig 4A glp-1 z-stack.tif]

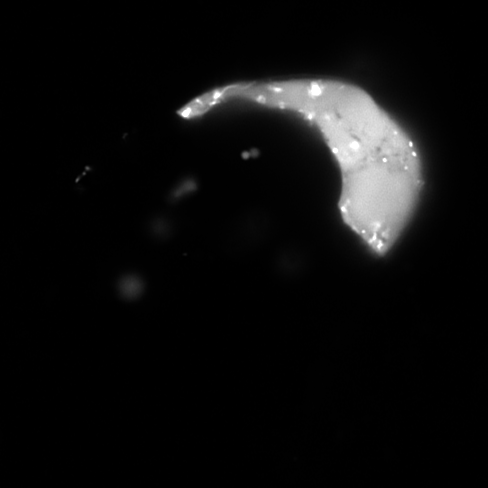

Supplement: Supplementary file 9 — Source data Fig. 4 [file 44318_2024_185_MOESM9_ESM.zip › 4A/Fig 4A WT shown slice.tif]

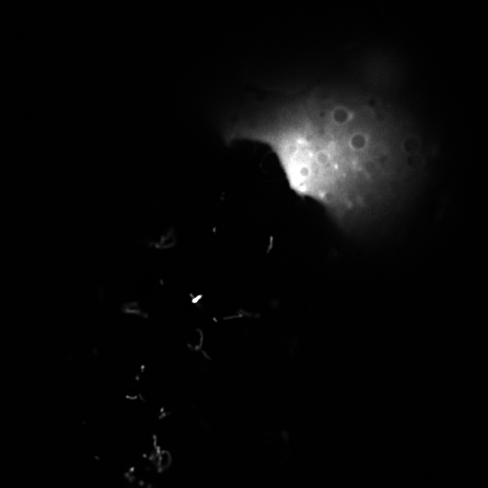

Supplement: Supplementary file 9 — Source data Fig. 4 [file 44318_2024_185_MOESM9_ESM.zip › 4A/Fig 4A WT z-stack.tif]
