# Supplementary material for: Germline loss in C. elegans enhances longevity by disrupting adhesion between niche and stem cells
Source: EMBO J. 2024 Jul 25;43(18):9. doi: 10.1038/s44318-024-00185-3 (PMC11405865; doi:10.1038/s44318-024-00185-3)
Supplement: Supplementary file 12 — Expanded View Figures [file 44318_2024_185_MOESM12_ESM.pdf]

Expanded View Figures

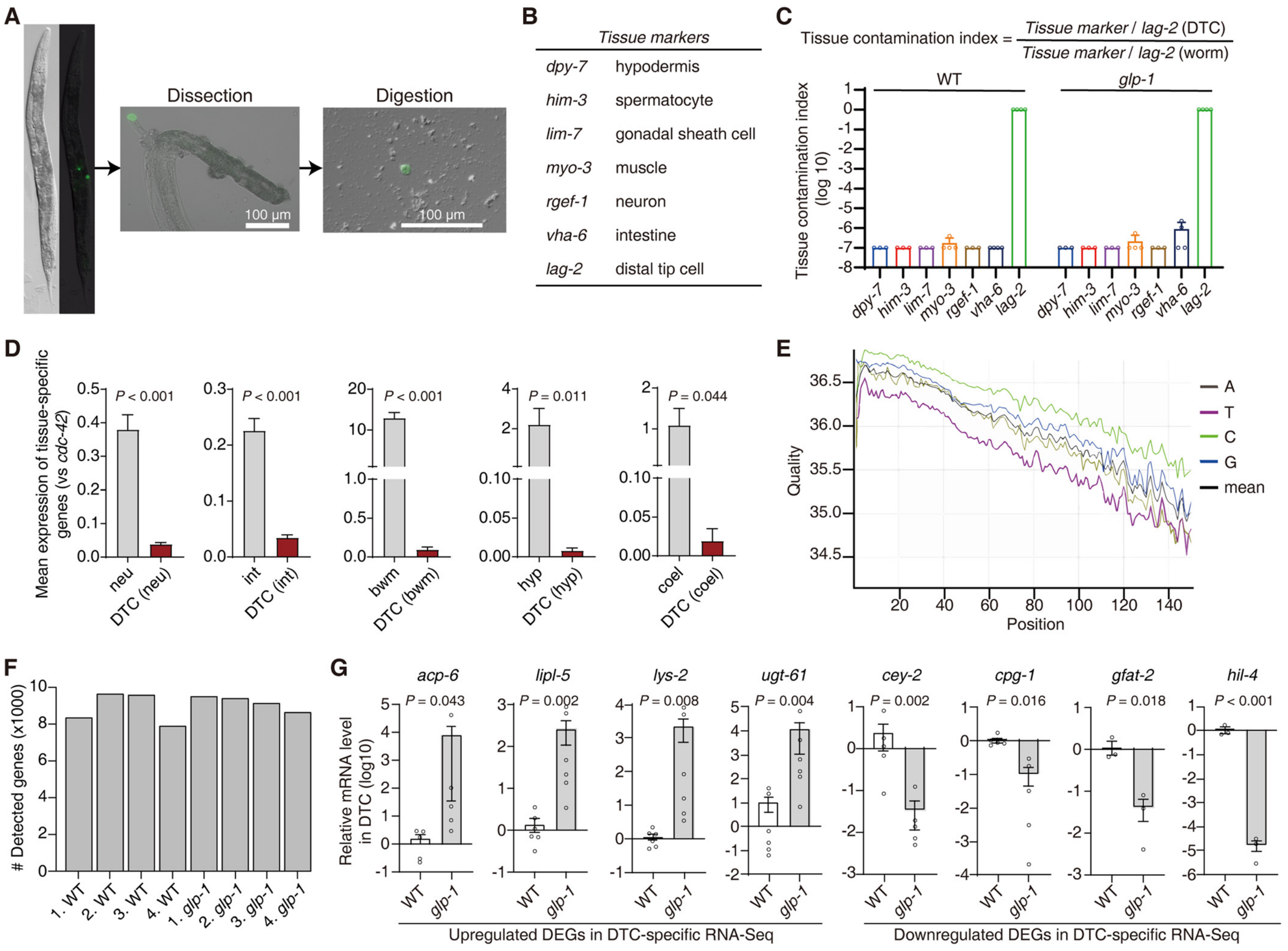

Figure EV1. RNA-Seq of the isolated DTCs from WT worms and *glp-1* mutants.

(**A**) The workflow of isolating GFP-labelled DTCs from worms. Scale bar: 100 μm. (**B**) A list of tissue marker genes. (**C**) RT-qPCR analysis for the purity of isolated DTCs subjected to RNA-Seq. When gene expression was below the detection limit, the corresponding tissue contamination index was set as 10^{-7}. At least 4 biological replicates were examined. Error bars: SD. (**D**) The expression of genes detected specifically in neuron (neu), intestine (int), body wall muscle (bwm), hypodermis (hyp), and coelomocyte (coel) by RNA-Seq (Wang et al, 2022) and in our DTC-specific RNA-Seq dataset. *cdc-42* serves as a reference gene for normalization. 345, 140, 33, 24, and 4 genes specifically detected in neu, int, bwm, hyp, and coel are analysed by their mean expression levels. Error bars: SEM. Unpaired *t*-test. At least 4 biological replicates were examined. (**E**) Reads quality of RNA-Seq on isolated DTCs. Quality shows the error rate of indicated bases. The higher the base quality value is, the less likely the base is mis-detected. The error rate corresponding to quality 30 is 99.9%, and 40 is 99.99%. (**F**) Detected genes in the indicated DTC samples. (**G**) RT-qPCR of isolated DTCs for the representative upregulated and downregulated genes identified by DTC-specific RNA-Seq. Unpaired *t*-test. At least 3 biological replicates were examined. Source data are available online for this figure.

© The Author(s)

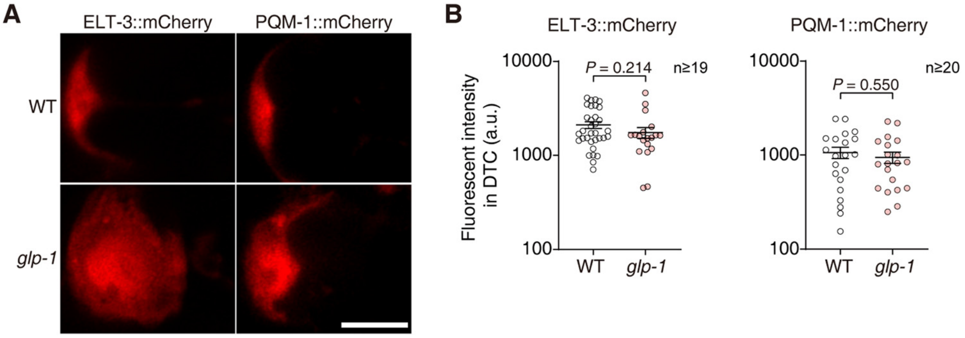

Figure EV2. The loss of germline does not change the expression of ELT-3 or PQM-1 in DTC.

(**A**) The expression of ELT-3::mCherry or PQM-1::mCherry in the DTC of indicated strains. Representative optical slices focusing on DTC soma are shown. Scale bar: 5 μm. (**B**) Quantification of the fluorescent intensity of ELT-3::mCherry and PQM-1::mCherry in the DTC of indicated strains. Error bars: SEM. Unpaired *t*-test. At least 19 biological replicates (DTCs) were examined. Source data are available online for this figure.

© The Author(s)

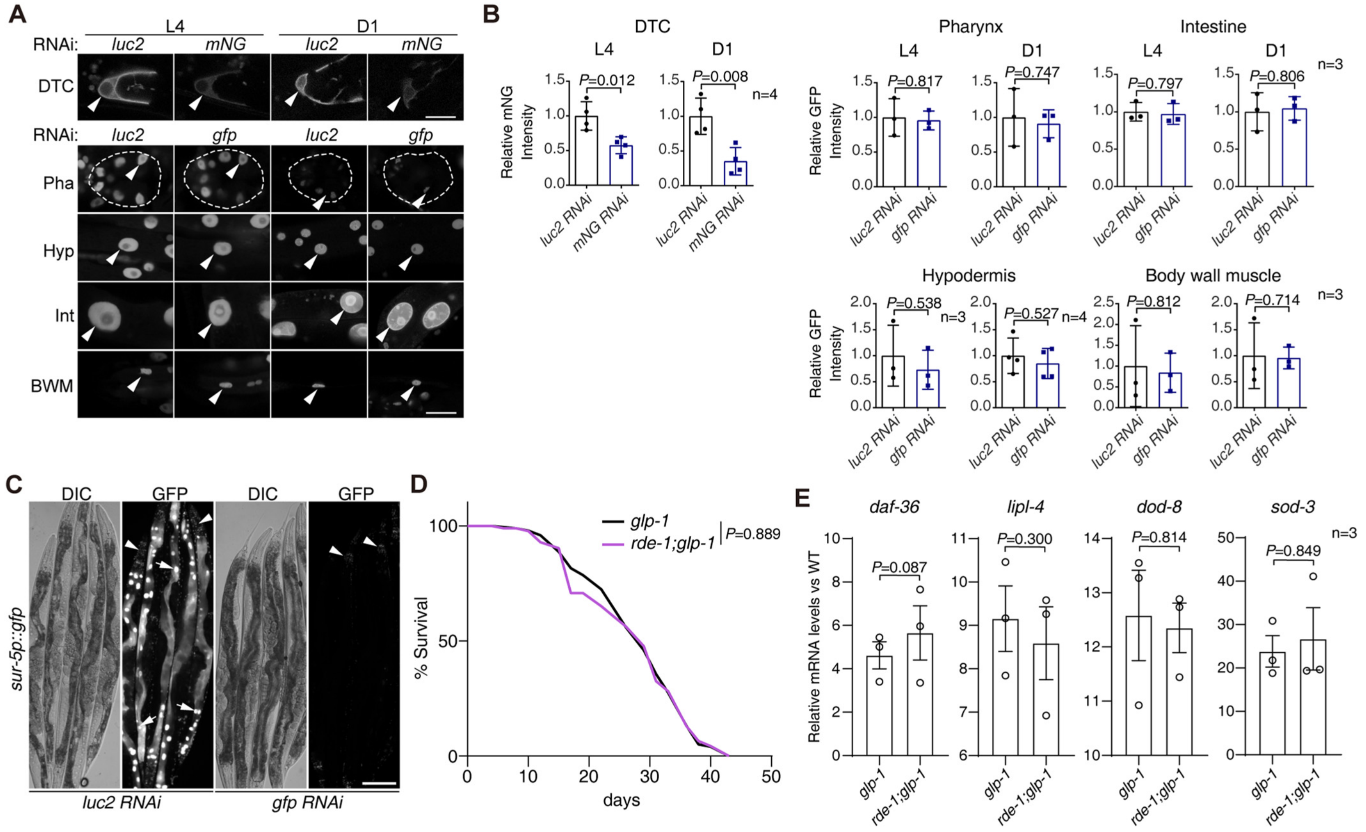

Figure EV3. The validation of the specificity of RNAi in DTC.

(**A**) Representative images of RNAi against mNG in DTC and GFP in other indicated tissues in the strain of DTC-specific RNAi. The strain for DTC-specific RNAi is a *rde-1* mutant with *rde-1* rescued in DTC via a single copy transgene, as reported by Sherwood lab. mNG was driven by the promoter of *lag-2*, whereas GFP was by the promoter of *sur-5*. Arrowheads denote the tissues of interest. L4: the 4th larval stage, D1: day 1 of adulthood. Scale bar: 10 µm. (**B**) The fluorescent intensity of mNG in DTC and GFP in other tissues upon indicated DTC-specific RNAi treatment. Error bars: SD. Unpaired *t*-test. At least 3 biological replicates were examined. (**C**) RNAi against *gfp* efficiently reduces the expression of *sur-5p::GFP* in WT worms. Note that the GFP signal in neurons are barely affected by *gfp* RNAi due to the insensitivity of RNAi in this tissue. Arrows denote intestine, arrowheads denote head neurons. Scale bar: 100 µm. (**D**) The survival curves of *glp-1* and *rde-1;glp-1* mutants. Mantel-Cox test. A representative biological replicate is shown for lifespan analyses. See source data for other biological replicates and detailed statistics. (**E**) The transcription of indicated genes in *glp-1* and and *rde-1;glp-1* mutants. Error bars: SEM. Unpaired *t*-test. 3 biological replicates were examined. The firefly luciferase gene, *luc2*, serves as the negative control in RNAi assays. Source data are available online for this figure.

© The Author(s)

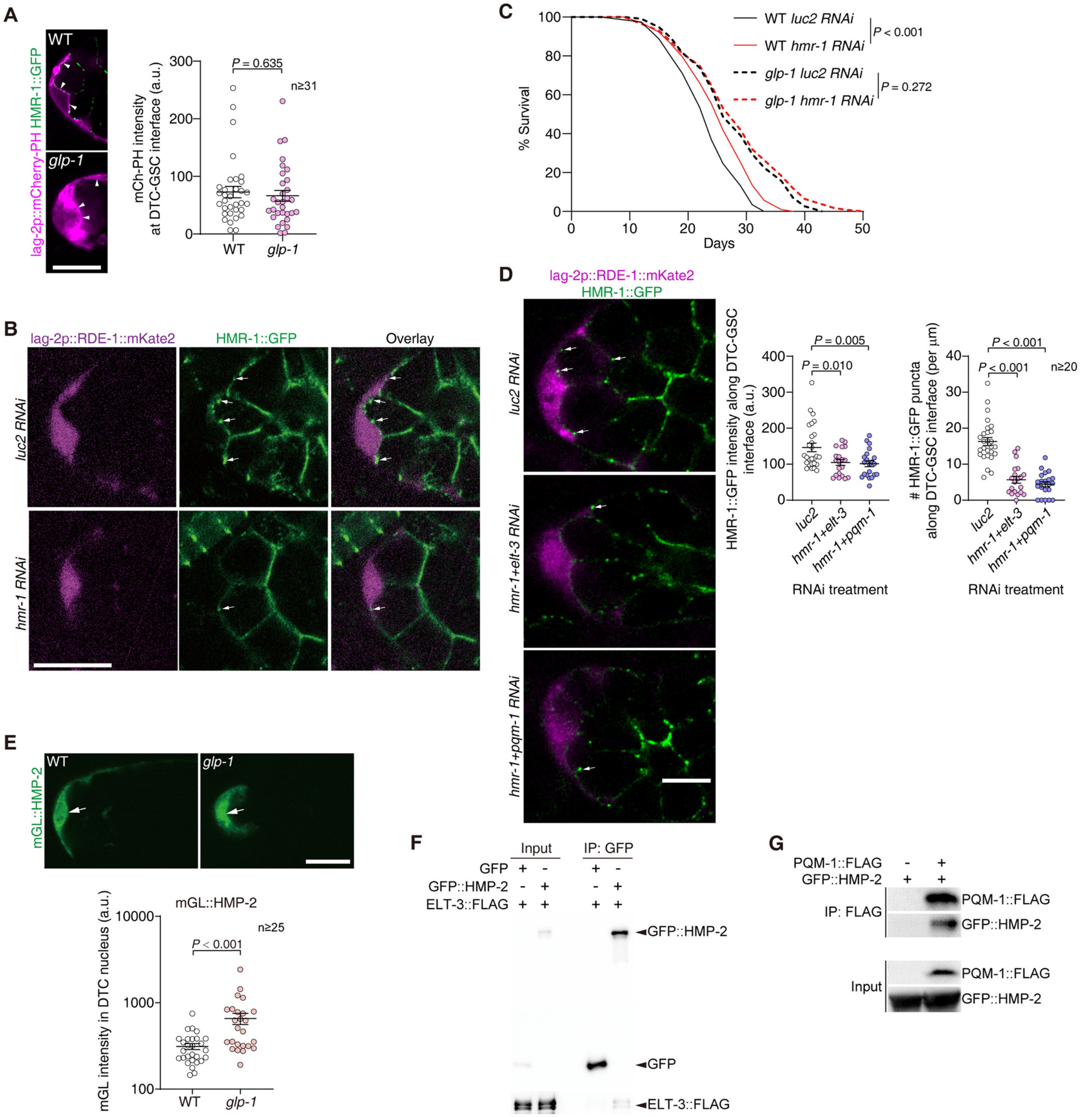

Figure EV4. The reduction of cell adhesions between DTC and GSC induces gonadal longevity.

(**A**) The membrane bound mCherry-PH is not decreased at DTC-GSC interface (arrowheads), unlike HMR-1::GFP labelled cell adhesions. Two optical slices from the Z-stack in Fig. 3B are shown. Unpaired *t*-test. Error bars: SEM. Scale bar: 10 µm. At least 31 biological replicates (DTCs) were examined. (**B**) DTC-specific RNAi against *hmr-1* reduced DTC-GSC adhesions (arrows) but not the adhesions in other germline cells. lag-2p::RDE-1::mKate2 labels DTC. Representative optical slices focusing on DTC soma are shown. Scale bar: 10 µm. (**C**) DTC-specific RNAi against *hmr-1* extends the lifespan of WT worms (8.7% extension in median lifespan) but not that of *glp-1* mutants. Mantel-Cox test. A representative biological replicate is shown. See source data for other biological replicates and detailed statistics. (**D**) HMR-1::GFP signal along the DTC-GSC interface and DTC-GSC adhesions (arrows) are significantly reduced upon the DTC-specific double RNAi against *hmr-1* and indicated GATA TFs (same as Fig. 3E, F). Representative optical slices focusing on DTC soma are shown. Scale bar: 5 µm. Unpaired *t*-test. Error bars: SEM. At least 20 biological replicates (DTCs) were examined. (**E**) The nuclear localization of mGreenLantern (mGL)-tagged HMP-2 (arrows) is increased in the DTC of *glp-1* mutants. Representative optical slices focusing on DTC soma are shown. Scale bar: 10 µm. Error bars: SEM. Unpaired *t*-test. At least 25 biological replicates (DTCs) were examined. (**F**, **G**). Co-immunoprecipitation of ELT-3::FLAG (**F**) or PQM-1::FLAG (**G**) with GFP::HMP-2 in HEK293T cells. The firefly luciferase gene, *luc2*, serves as the negative control in RNAi assays. Source data are available online for this figure.

 © The Author(s)

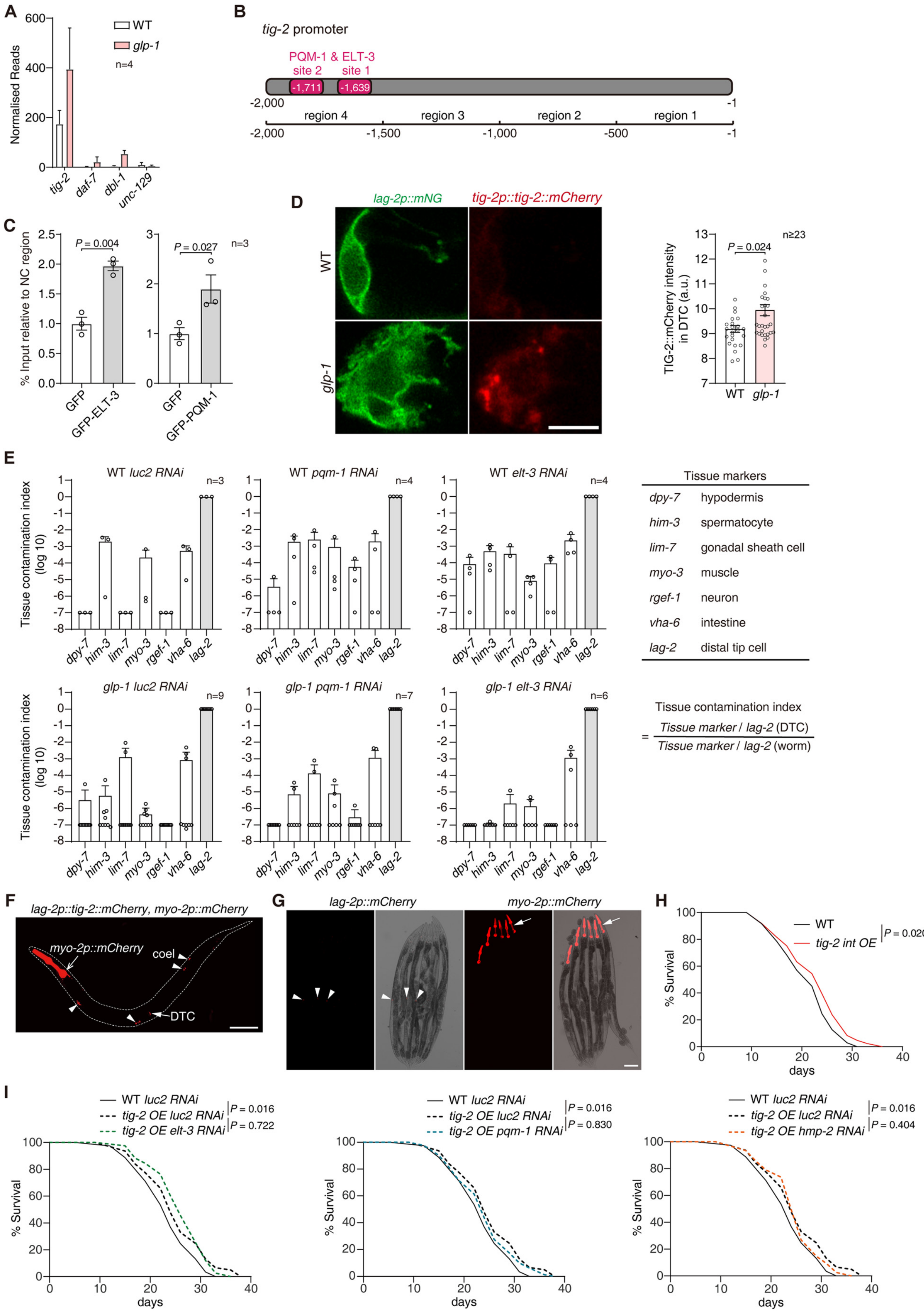
A
Normalised Reads
WT
glp-1
n=4
tig-2
daf-7
dbl-1
unc-129
B
tig-2 promoter
PQM-1 & ELT-3
site 2
site 1
-1,711
-1,639
-2,000
-1
region 4
region 3
region 2
region 1
-2,000
-1,500
-1,000
-500
-1
C
% Input relative to NC region
P = 0.004
P = 0.027
n=3
GFP
GFP-ELT-3
GFP
GFP-PQM-1
D
lag-2p::mNG
tig-2p::tig-2::mCherry
WT
glp-1
TIG-2::mCherry intensity in DTC (a.u.)
P = 0.024
n≥23
WT
glp-1
E
Tissue contamination index (log 10)
WT luc2 RNAi
n=3
WT pqm-1 RNAi
n=4
WT elt-3 RNAi
n=4
glp-1 luc2 RNAi
n=9
glp-1 pqm-1 RNAi
n=7
glp-1 elt-3 RNAi
n=6
dpy-7
him-3
lim-7
myo-3
rgef-1
vha-6
lag-2
Tissue markers
dpy-7 hypodermis
him-3 spermatocyte
lim-7 gonadal sheath cell
myo-3 muscle
rgef-1 neuron
vha-6 intestine
lag-2 distal tip cell
Tissue contamination index
= Tissue marker / lag-2 (DTC) / Tissue marker / lag-2 (worm)
F
lag-2p::tig-2::mCherry, myo-2p::mCherry
myo-2p::mCherry
coel
DTC
G
lag-2p::mCherry
myo-2p::mCherry
H
% Survival
days
WT
tig-2 int OE
P = 0.020
I
WT luc2 RNAi
tig-2 OE luc2 RNAi
tig-2 OE elt-3 RNAi
P = 0.016
P = 0.722
WT luc2 RNAi
tig-2 OE luc2 RNAi
tig-2 OE pqm-1 RNAi
P = 0.016
P = 0.830
WT luc2 RNAi
tig-2 OE luc2 RNAi
tig-2 OE hmp-2 RNAi
P = 0.016
P = 0.404
% Survival
days

Figure EV5. The induction of *tig-2* in DTC upon germline removal promotes longevity.

(**A**) The expression level of the four detected TGF-β ligand genes in DTC-specific RNA-Seq. *n*: the number of biological replicates. Each sample of one biological replicate contains around 12 DTCs. Error bars: SEM. (**B**) A diagram showing the binding sites of ELT-3 and PQM-1 in *tig-2* promoter. (**C**) HEK293T cells co-expressing indicated GFP proteins and a luciferase reporter driven by *tig-2* promoter were subjected to ChIP analysis. Note that *tig-2* promoter is co-immunoprecipitated with GFP-ELT-3 and GFP-PQM-1. The 3′-UTR of luciferase serves as the negative control. 3 biological replicates were examined. Error bars: SEM. (**D**) The expression of TIG::mCherry in DTC is increased in *glp-1* mutants. lag-2p::mNG labels DTC. Representative optical slices focusing on DTC soma are shown. Scale bar: 5 μm. At least 23 biological replicates (DTCs) were examined. Error bars: SEM. (**E**) Isolated DTCs for RT-qPCR of *tig-2* were analysed for their purity. When gene expression was below the detection limit, the corresponding tissue contamination index was set as 10^{-7}. At least 3 biological replicates were examined. Error bars: SD. (**F**, **G**) The expression of TIG-2::mCherry (**F**) and mCherry (**G**) by indicated promoters. The strong red fluorescence in the pharynx (**F**) is from the injection marker, *myo-2p::mCherry*. Note in (**F**) that TIG-2::mCherry is detected not only in DTC (arrow) but also in coelomocytes (arrowheads). mCherry driven by *lag-2* or *myo-2* promoter was not detected in coelomocyte. Arrowheads in (**G**) denote DTC, whereas arrows in (**G**) pharynx. Scale bar: 100 μm. (**H**) The artificial overexpression of *tig-2* in intestine extends the lifespan of WT worms (9.1% extension in median lifespan). (**I**) DTC-specific RNAi against *elt-3*, *pqm-1*, or *hmp-2* does not suppress the longevity induced by overexpressing *tig-2* in DTC (10.3% extension in median lifespan). The firefly luciferase gene, *luc2*, serves as the negative control in RNAi assays. Unpaired *t*-test in (**C**) and (**D**), Mantel-Cox test in (**H**) and (**I**). A representative biological replicate is shown for lifespan analyses in (**H**) and (**I**). See source data for other biological replicates and detailed statistics of (**H**) and (**I**). Source data are available online for this figure.

© The Author(s)
